# Neurosurgical Management of Central Nervous System Lymphoma: Lessons Learnt from a Neuro-Oncology Multidisciplinary Team Approach

**DOI:** 10.3390/jpm13050783

**Published:** 2023-04-30

**Authors:** Maria Alexandra Velicu, Jose Pedro Lavrador, Naomi Sibtain, Francesco Vergani, Ranjeev Bhangoo, Richard Gullan, Keyoumars Ashkan

**Affiliations:** 1 Department of Neurosurgery, King’s College Hospital NHS Foundation Trust, London SE5 9RS, UK; 2 Department of Neuroradiology, King’s College Hospital NHS Foundation Trust, London SE5 9RS, UK

**Keywords:** CNS lymphoma, DLBCL, PCNSL, lymphoma CNS relapse, extranodal lymphoma

## Abstract

Central nervous system lymphoma (CNSL) represents one of the most aggressive forms of extranodal lymphoma. The gold standard for CNSL diagnosis remains the stereotactic biopsy, with a limited role for cytoreductive surgery that has not been supported by historical data. Our study aims to provide a comprehensive overview of neurosurgery’s role in the diagnosis of systemic relapsed and primary CNSL, with an emphasis on the impact on management and survival. This is a single center retrospective cohort study with data collected between August 2012 and August 2020, including patients referred with a potential diagnosis of CNSL to the local Neuro-oncology Multidisciplinary Team (MDT). The concordance between the MDT outcome and histopathological confirmation was assessed using diagnostic statistics. A Cox regression is used for overall survival (OS) risk factor analysis, and Kaplan–Meier statistics are performed for three prognostic models. The diagnosis of lymphoma is confirmed in all cases of relapsed CNSL, and in all but two patients who underwent neurosurgery. For the relapsed CNSL group, the highest positive predictive value (PPV) is found for an MDT outcome when lymphoma had been considered as single or topmost probable diagnosis. Neuro-oncology MDT has an important role in establishing the diagnosis in CNSL, not only to plan tissue diagnosis but also to stratify the surgical candidates. The MDT outcome based on history and imaging has good predictive value for cases where lymphoma is considered the most probable diagnosis, with the best prediction for cases of relapsed CNSL, questioning the need for invasive tissue diagnosis in the latter group.

## 1. Introduction

Diffuse large B-cell lymphoma (DLBCL) represents the most frequent form of Non-Hodgkin Lymphoma (NHL), affecting 25–58% of the cases, and central nervous system (CNS) relapse remains one of the adverse complications of DLBCL [1]. DLBCL is responsive to chemotherapy and the introduction of Rituximab has improved treatment outcomes, with 60% of the patients achieving long-term progression-free survival [1,2]. The gold standard prior to Rituximab approval was CHOP (cyclophosphamide, doxorubicin, vincristine, prednisone) [3], and the FDA approved Rituximab as the first-line treatment for DLBCL in 2006 when three phase III trials demonstrated its benefit when added to classical CHOP treatment, with a 2 and 5-year survival rates of 70% and 69% for the R-CHOP, versus 57% and 40% for CHOP alone [3]. Secondary (relapsed) CNS involvement occurs in up to 5% of DLBCL cases [4] and often within the first 2 years of completion of primary treatment [2], with poor outcomes and a median survival from the diagnosis of 2–5 months [1,5]. Despite improvements in the treatment of DLBCL, CNS recurrence and its prophylaxis represent an unmet need, with no consensus achieved to this date [6,7]. Primary CNS lymphoma (PCNSL) accounts for 1–2% of all primary CNS tumours with a 5-year survival of 15–30% [8,9]. For PCNSL, high-dose methotrexate remains the cornerstone of the treatment, in combination with other agents and whole brain radiotherapy, but the optimal chemotherapeutic combination as well as the role of radiotherapy remain controversial [10,11].

The incidence of CNS lymphoma (CNSL) has increased over the past decades in immunocompetent patients [12]. Furthermore, similar to other tumours, the understanding of the molecular pathogenesis of DLBCL has constantly evolved, and several biologic features have been identified as prognostic factors or as disease characteristics relevant to treatment. High-grade B-cell lymphoma with translocations of MYC and BCL2 and/or BCL6 genes has been associated with a worse prognosis [5,13], whereas mutations such as MYD88 and CD79B have been frequently described in PCNSL [11]. In cases of relapsed CNSL, primary lymphoma locations such as testes, breast, and uterine DLBCL have a high prevalence of the non-germinal center B-cell (non-GCB) phenotype and the MYD88/CD79B-mutated genotype with a more reserved prognosis [13]. Other primary locations such as craniofacial, thyroid, localized bone, or gastric-lack MYD88 mutations, share an improved prognosis and low CNS recurrence rates [13]. 

The importance of correct diagnosis and the relevance of molecular markers to treatment and prognosis are increasingly driving the requests for neurosurgical input. Historically this equates to an early neurosurgical intervention to obtain a histopathological diagnosis [14]. However, in the recent decade, the experience built by the neurosurgical neuro-oncology MDT warrants a rethink about the contribution of neurosurgery to the management of lymphoma over and above a purely technical one to perform a biopsy. The initial assessment by a neuro-oncology MDT aims to provide differential diagnoses and management based on clinical (including ophthalmic and CSF analysis) and imaging characteristics, as well as an assessment of the indication for surgery and overall surgical risks. From a clinical point of view, a previous diagnosis of systemic lymphoma or physical finding of lymphadenopathy are relevant. Ophthalmic assessments show abnormalities in 15–25% of patients [15], whilst CSF might be abnormal in 16–22% of patients with CNSL [16,17]. In terms of imaging, the majority of both PCNSL (in immunocompetent patients) and relapsed CNSL have characteristic features on both conventional (CT, MRI) and advanced imaging modalities (diffusion weighted imaging—DWI, perfusion MR and MR spectroscopy) [12,18,19,20]. However, several of these imaging characteristics may overlap with other neoplasms such as high-grade gliomas, metastases and meningiomas as well as non-neoplastic conditions such as granulomatous disease. A smaller proportion of cases, including those seen in the setting of immunocompromise, show atypical imaging features which are less specific and overlap more substantially with a wider range of neoplastic and non-neoplastic processes, including atypical infections and tumefactive demyelinating plaques. Typical imaging features of PCNSL reflect the high nuclear:cytoplasmic ratio and are shown as solitary or multiple parenchymal lesions that are iso- or hyperdense on CT, T1 hypo- or isointense and T2 isointense on MRI, and with fairly avid homogeneous enhancement [12,20,21,22]. Lesions are typically supratentorial in location and the vast majority lie at the parenchymal/CSF interface (either pial or ventricular) [12,20]. With regard to relapsed CNSL, approximately two-thirds of patients present with leptomeningeal disease that often involves the cranial nerves and spinal nerve roots, whilst one-third present with parenchymal disease [20,23]. On advanced imaging, lesions show a marked diffusion restriction with lower apparent diffusion coefficient (ADC) values compared with those seen with high-grade gliomas and metastases [19,24]. Markedly elevated choline and lipid peaks on MR spectroscopy can help differentiate PCNSL from tumefactive plaques or abscesses, although this spectroscopic pattern may overlap with high-grade gliomas [7,10,24,25]. Since tumour neovascularisation is absent in PCNSL, a perfusion MRI may show lower mean relative cerebral blood volumes compared with high-grade gliomas [26].

In terms of the MDT contribution in evaluating surgical risks, a higher incidence of CNSL among the elder population with co-morbidities, the increased associated morbidity due to their location and high sensitivity of lymphoma cells to corticosteroid-induced apoptosis, which can alter their morphology and lead to non-diagnostic biopsies that would delay further adjuvant treatment [25,27,28], warrant a meticulous surgical risk assessment with an emphasis on the careful evaluation of the added diagnostic value [8]. The aim of this study is to evaluate the role of neurosurgery in the management of relapsed systemic and primary CNS lymphoma, especially where a biopsy is indicated and where the risks of surgery outweigh the benefits. Given the paucity of UK data on survival of patients with PCNSL, such data are also reported.

## 2. Materials and Methods

This is a single-centre retrospective cohort study with data collected between August 2012 and August 2020. All patients who were referred with a potential diagnosis of CNS lymphoma or diagnosed with CNS lymphoma after being referred to the neuro-oncology MDT at a single quaternary neurosurgical centre are included in the study. The members of the neuro-oncology MDT are neurosurgeons, neuroradiologists, neuro-oncologists, neurologists, specialist nurses, speech and language therapists and physiotherapists. Definitive diagnosis is established either by biopsying the intracranial lesion (surgical group) or through non-neurosurgical procedures such as ophthalmic assessment, lumbar puncture, or lymph node biopsy (non-surgical group). Data were also collected on those patients referred with a potential diagnosis of lymphoma in whom non- neurosurgical procedures failed to establish the diagnosis and who were not deemed suitable for a neurosurgical procedure (added to non-surgical group under True-Not confirmed). Both patients with relapsed systemic lymphoma and primary CNS lymphoma are included. The exclusion criteria are: suspected or confirmed metastatic spinal cord lymphoma, referrals with no MDT pre-operative clinical/radiological diagnosis available and incomplete referrals. Data were processed using IBM SPSS^®^ version 25.

A neuro-oncology MDT outcome analysis was performed for the entire surgical and non-surgical cohorts, and data was further categorised as relapsed or primary CNSL for the same analysis. The concordance between the diagnosis suggested by the neuro-oncology MDT prior to surgery and tissue histopathological results was evaluated using non-parametric tests. Diagnostic statistics were calculated for each group and diagnostic category, and a likelihood ratio reported. A logistic regression was employed to analyse the contribution of several factors in the assessment of the surgical risk: age, Eastern Cooperative Oncology Group performance status (ECOG PS), Glasgow Coma Scale (GCS) score, tumour location, multifocal or meningeal involvement and comorbidities. The locations of the lesions are divided into deep and superficial: the white matter lesions located around the basal ganglia, brainstem or infraventricular are categorised as deep, and all other locations as superficial, including cerebellum [8,21,24,25].

Overall survival (OS) was calculated as the time between the diagnosis of CNSL recurrence or PCNSL and the time of death. The impact of various risk factors on the OS was calculated for the relapsed CNSL, PCNSL and the entire cohort using the Cox regression, and the results displayed for both univariate and multivariate regression. All the parameters were recorded from the time of the neurosurgical referral. Additionally, two known prognostic scores for PCNSL were used (Memorial Sloan Kettering Cancer Center-MSKCC and Nottingham/Barcelona-NB) and compared with a newly proposed score (Taipei). The prognostic scores were applied to both groups and the entire cohort, and the statistical significance of their impact on the OS was determined prior to the survival analysis using a Cox regression. A further analysis of the prognostic scores was performed using ROC curves. A Kaplan–Meier survival analysis was performed, with mean and median OS summarised for each group and prognostic score, along with the graphic representation of the survival curves. Missing values are excluded from the analysis. A *p* < 0.05 is considered statistically significant.

## 3. Results

### 3.1. Surgical Cases vs. Non-Surgical Cases

A total of 129 patients are included in the study. 76 patients underwent surgery to obtain tissue from the intracranial lesion as recommended by the neuro-oncology MDT, and 53 were not considered neurosurgical candidates. Most of the patients from both surgical and non-surgical groups have ages below 80 years, with a trend towards older age observed in the non-surgical group (93.4% vs. 77.3%). The majority of the surgical cases has a good PS (ECOG PS < 2, 85.5%) as compared with the non-surgical group (33.9%), as well as high GCS score (15/15, 80.2% vs. 67.9%). Neurological deficits are found in similar proportions in both groups (64.4% vs. 69.8%), whereas seizures and cognitive impairment re more frequent findings at presentation in the surgical group (11.8% vs. 1.8% and 34.2% vs. 26.4%). Within the surgical and non-surgical cases, respectively, 77.6% and 88.6% presented with symptoms lasting less than one month. (Table 1).

Based on the clinical and radiological features, lymphoma was considered among the differential diagnoses by the neuro-oncology MDT in 80.2% (61/76) of the patients who underwent surgery, and a single or top differential diagnosis of lymphoma was suggested before the procedure by the MDT in 60.5% (46/76) of the cases. (Table 2) In 44 out of 46 surgical patients where lymphoma was considered by the MDT as the single or top differential diagnosis, the histopathological diagnosis confirmed lymphoma, indicating a 95.6% positive predictive value (PPV) for the neuro-oncology MDT outcome (likelihood ratio χ^2^ = 2.044, *p* = 0.153). Out of 61 patients who underwent surgery and had lymphoma as single, first or among the differential diagnoses, 59 had a confirmed histopathological diagnosis of lymphoma, with a PPV of 96.7% for the neuro-oncology MDT outcome (likelihood ratio χ^2^ = 0.893, *p* = 0.345). Lymphoma was not considered among the differential diagnoses for 15/76 patients who underwent surgery, and subsequently had a histopathological confirmation of lymphoma. 

Within the non-surgical group, 47/53 (88.6%) had lymphoma as single or top differential diagnosis, and 10/53 (18.9%) had the diagnosis verified, either confirmed (8/10) or excluded (2/10) by other diagnostic procedures. (Table 2).

### 3.2. Relapsed vs. Primary CNS Lymphoma

A total of 36 patients had known previous systemic or newly diagnosed systemic lymphoma with CNS involvement, and 15/36 underwent a neurosurgical procedure. Among the surgical patients, 12/15 had lymphoma as single or top differential diagnosis, with a PPV of 100% for the neuro-oncology MDT diagnosis. All patients with relapsed CNSL who underwent surgery (15/36) had the diagnosis of lymphoma confirmed on histopathology. (Table 3).

Within the PCNSL group, 61/93 patients underwent surgery. The diagnosis was confirmed for 46/48 surgical patients who had lymphoma among the MDT’s differential diagnoses, with a PPV of 95.8% (likelihood ratio χ2 = 0.977, *p* = 0.323). In 32 out of 34 cases where lymphoma was the single or top differential pre-operative diagnosis, this was confirmed by the histological analysis, with a PPV of 97% (likelihood ratio χ2 = 2.392, *p* = 0.122). (Table 3) The diagnosis of lymphoma was excluded in 2 non-surgical (assessed by CSF analysis) and 2 surgical PCNSL cases in whom other diagnoses were made. (Table 3 and Table 4).

The analysis of the clinical factors evaluated in the neuro-oncology MDT showed that age < 80 years (OR 10.82 [2.31, 50.75], *p* = 0.003) and ECOG PS < 2 (OR 14.47 [5.89, 35.55], *p* < 0.001) are clinical characteristics in favour of a neurosurgical management. The other factors (GCS score, location, multifocal/meningeal involvement and >1 comorbidity) do not reach statistical significance as independent factors influencing the neurosurgical management. However, having more than two of the six risk factors is associated with non-surgical management (OR 4.62 [2.09, 10.23], *p* < 0.001) (Table 5).

**Table 5:** Summary of the clinical factors contributing to the surgical risk evaluation.

Risk Factor	OR (CI 95%)	*p* Value	R^2^
NSL-surgical vs. non-surgical			
Age < 80	10.82 (2.31, 50.75)	0.003	0.133
ECOG PS < 2	14.47 (5.89, 35.55)	<0.001	0.377
GCS < 15	1.84 (0.76, 4.44)	0.170	0.021
Location	1.71 (0.82, 3.56)	0.149	0.022
Multifocal/meningeal	1.71 (0.80, 3.61)	0.161	0.022
>1 comorbidity *	1.62 (0.66, 3.96)	0.288	0.013
<2 risk factors †	4.62 (2.09, 10.23)	<0.001	0.159

* Assessed using the 11-item modified frailty index (mFI): diabetes mellitus; functional status 2 (not independent); chronic obstructive pulmonary disease or pneumonia; congestive heart failure; myocardial infarction; percutaneous coronary intervention, stenting, or angina; hypertension requiring medication; peripheral vascular disease or ischemic rest pain; impaired sensorium; transient ischemic attack or cerebrovascular accident; and cerebrovascular accident with neurological deficit. The functional status has been examined as independent risk factor and not as part of the comorbidity index. † Assessed using a 6-item scale derived from the previously examined factors: PS < 2, age < 80, GCS < 15, location, multifocal/meningeal involvement and >1 comorbidity. Note: OR-odds ratio; R^2^—coefficient of determination.

### 3.3. Surgical Complications

A total of 11 out of 76 patients (14.5%) presented post-operative complications, and the most frequent was sepsis (*n* = 3, 3.9%). Haemorrhage at the surgical bed site occurred in 2 out of 76 patients; none required surgical evacuation. Early complications included increased seizure frequency (*n* = 1), hydrocephalus (*n* = 1), worsening neurological deficit (*n* = 1) and pulmonary embolus for one of the patients with sepsis (*n* = 1). Late complications were subdural and subgaleal collections requiring the placement of a ventriculo-peritoneal shunt (*n* = 2) and chronic subdural haemorrhage (*n* = 1). Of all patients who had a biopsy (*n* = 66), 5 required a second procedure due to an inconclusive tissue specimen (7.5%) thought to be related to an insufficient time off steroids (at least 10 days) before the biopsy. 

### 3.4. Molecular Characteristics

Of all DLBCL cases, ABC-like DLBCL is the predominant subtype among PCNSL (84% ABC-like DLBCL vs. 16% GC-like subtype), whereas relapsed CNSL cases show a more heterogenous distribution (45.5% ABC-like DLBCL vs. 54.5% GC-like DLBCL). The vast majority of CNSL cases are CD23 negative (80% PCNSL, 100% relapsed CNSL) as well as CD138 negative (91.7% PCNSL, 60% relapsed CNSL), and cyclin D1 negative (88.5% PCNSL, 80% relapsed CNSL). Both PCNSL and relapsed CNSL show predominant CD3 positivity (73.9% PCNSL, 100% relapsed CNSL) and CD5 negativity (100% PCNSL, 75% relapsed CNSL). CD10 positivity is found in 70% cases of relapsed CNSL, and only in 37.8% of PCNSL cases. The majority of PCNSL cases are CD30 negative (60%) with only one positive case in the relapsed CNSL group. Epstein-Barr virus (EBV) encoded small RNAs positivity is present in 33.3% of PCNSL cases and 12.5% of relapsed CNSL cases. 

### 3.5. Risk Factors and Outcome

For the cases of relapsed CNSL, the univariate regression identifies several statistically significant risk factors for the OS: CNS prophylaxis (*p* = 0.002), radiotherapy during initial treatment (*p* = 0.021) and <3 cycles of chemotherapy during the CNS relapse treatment (*p* = 0.022). Only CNS prophylaxis remains statistically significant in the multivariate analysis (*p* = 0.022). Both the univariate and multivariate regressions find the same statistically significant risk factors for the OS for the PCNSL group: age (*p* = 0.013 and *p* = 0.015), ECOG PS (*p* < 0.001 and *p* = 0.001), albumin levels (*p* = 0.020 and *p* = 0.021), <3 cycles of chemotherapy during the CNS relapse treatment (*p* = 0.002 and *p* = 0.005) and autologous stem cell transplantation (ASCT) (*p* = 0.010 and *p* = 0.035). The analysis for the entire CNSL cohort shows similar results to those found for the PCNSL group with ECOG PS (*p* < 0.001 and *p* < 0.001), albumin levels (*p* = 0.007 and *p* = 0.017), <3 cycles of chemotherapy (*p* < 0.001 and *p* = 0.026) and ASCT (*p* = 0.001 and *p* = 0.020) representing statistically significant risk factors for OS in both the univariate and multivariate regression. (Supplementary Material, Tables S1 and S2).

**Figure 1:**
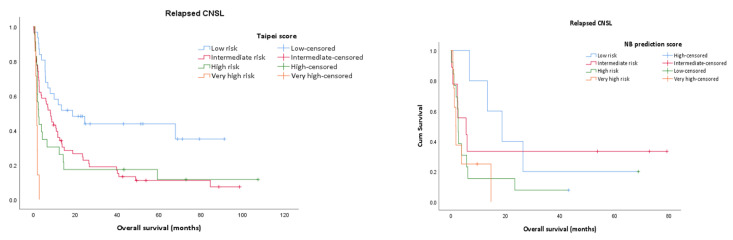

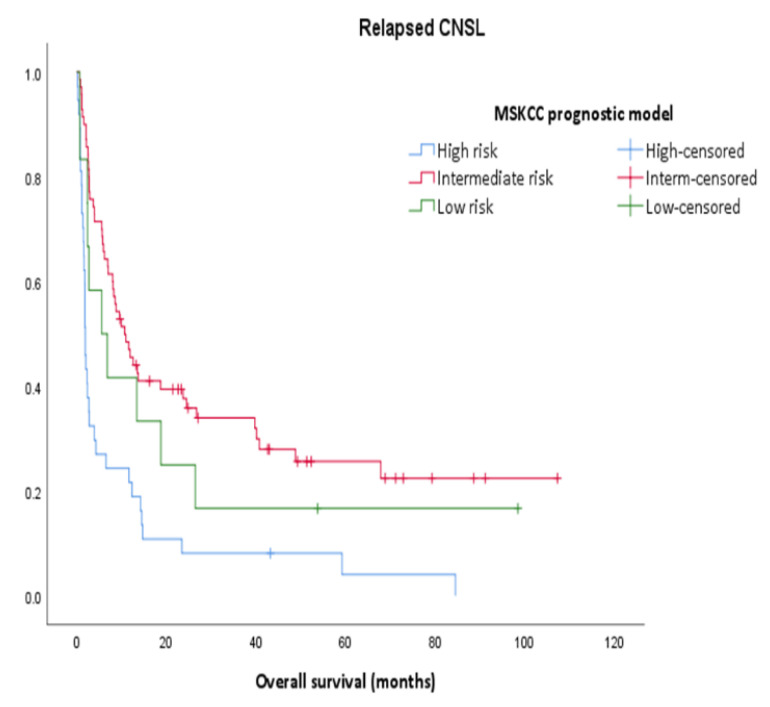
Kaplan–Meier survival analysis for relapsed CNSL for each prognostic score (MSKCC prognostic score, Taipei score, NB prediction score).

**Figure 2:**
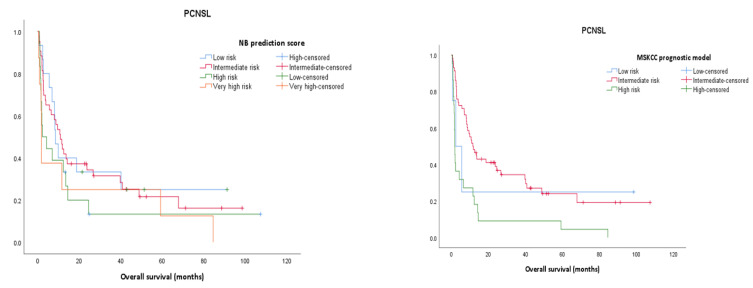

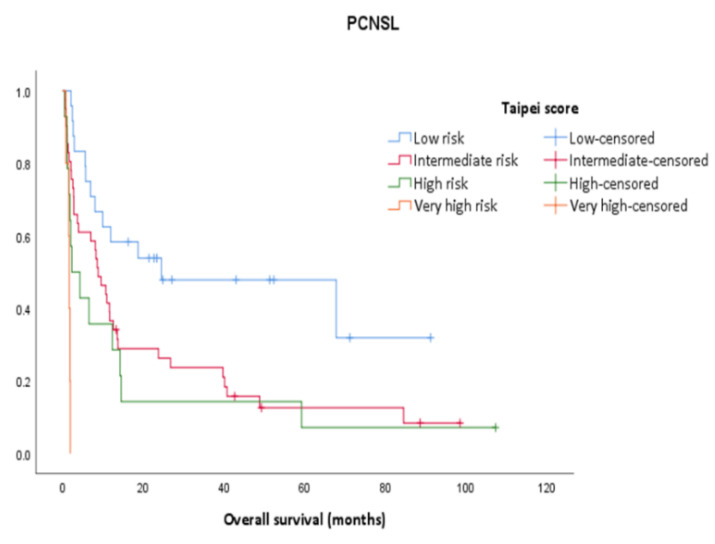
Kaplan–Meier survival analysis for PCNSL for each prognostic score (MSKCC prognostic score, Taipei score, NB prediction score).

**Figure 3:**
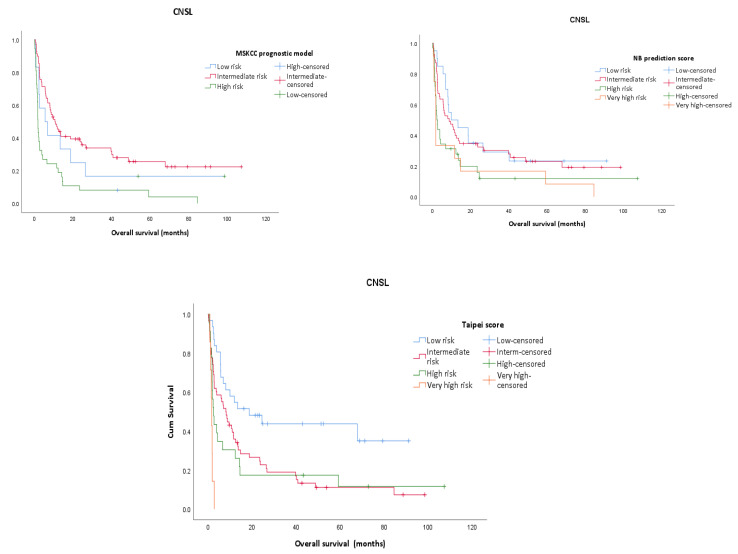
Kaplan–Meier survival analysis for the entire CNSL cohort for each prognostic score (MSKCC prognostic score, Taipei score, NB prediction score).

**Figure 4:**
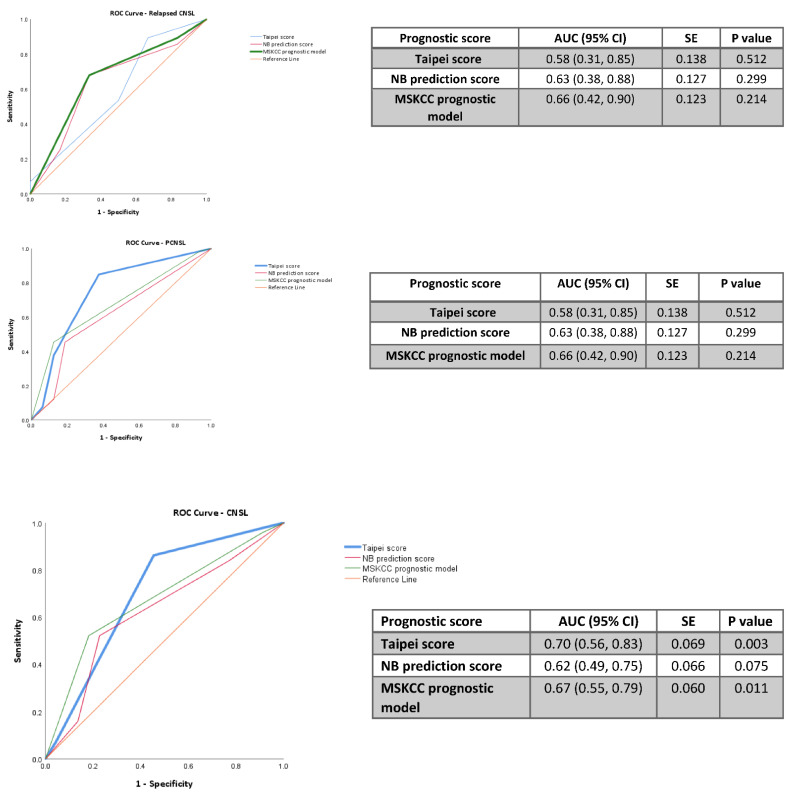
Receiver Operating Characteristic (ROC) Curves for each group (Relapsed CNSL, PCNSL and the entire cohort). ROC curves are followed by a table displaying the ROC analysis results for each prognostic score. Note: AUC, area under the curve; SE, standard error.

Within the relapsed CNSL group, the 3-year survival is 14.3%, whilst it reaches 23.9% for the PCNSL group. The assessment of prognostic scores shows that for relapsed CNSL none of the prognostic scores are significantly associated with the OS; however, the MSKCC prognostic model has better results (*p* = 0.071, results not shown) and it is also confirmed by the ROC curve (Figure 1 and Figure 4). Using the MSKCC prognostic score, the mean and median OS (95% CI) expressed in months for patients in the low, intermediate, and high-risk groups are 17.53 (0.00–38.56) vs. 2.36 (0.00–14.81), 30.64 (10.43–50.85) vs. 6.80 (0.00–26.68) and 6.98 (2.04–11.93) vs. 2.36 (1.07–3.66). For PCNSL, all three prognostic scores are statistically significantly associated with the OS and only two of them re confirmed by the ROC curve, with the Taipei score showing the best result (*p* = 0.001) (Supplementary Material, Table S3) (Figure 2 and Figure 4). The mean and median OS in months (95% CI) for the Taipei score for the low-, intermediate-, high- and very-high-risk groups are 48.90 (30.97–67.83) vs. 67.80 (2.88–132.71), 24.80 (14.35–35.25) vs. 9.53 (6.00–13.06), 7.98 (1.86–14.10) vs. 2.06 (0.99–3.13) and 19.13 (0.00–50.66) vs. 1.63 (1.23–2.03). For the entire CNSL cohort, all 3 prognostic scores are statistically significantly associated with the OS, and the Taipei score is the best predictor (Supplementary Materials, Table S3) (Figure 3 and Figure 4). The mean and median OS in months (95% CI) for the Taipei score for the low-, intermediate-, high- and very-high-risk groups are 48.32 (32.20–64.44) vs. 67.80 (0.00–145.32), 22.28 (13.37–31.20) vs. 8.53 (6.47–10.59), 11.19 (4.94–17.44) vs. 2.66 (1.85–3.48) and 14.83 (0.00–39.04) vs. 1.63 (1.17–2.09).

## 4. Discussion

Diagnosis of CNSL remains a challenge and accurate diagnosis is needed, especially given the impact of molecular markers on therapy and prognosis. Neurosurgery to obtain diagnostic tissue from intracranial lesions, however, carries risks, particularly in patients in older age group who have poor PS, multiple co-morbidities and deep-seated lesions, as is often the case in the cohort of patients evaluated for CNSL. Careful patient selection is therefore warranted. In the past decade, restructuring the surgical neuro-oncology services into MDTs has allowed the accrual of experience in the management of these lesions.

Despite the diagnostic challenges posed by patients with a form of lymphoma in their differential diagnosis, our findings have shown a high positive predictive value (≥95%) of the neuro-oncology MDT outcome when lymphoma is considered high on differential diagnosis. The PPV reaches 100% in relapsed CNSL when the MDT put lymphoma high up in the differential diagnosis, questioning the need to put this cohort of patients through the risk of neurosurgery for the confirmation of the diagnosis. In terms of overall surgical risk stratification, independent risk factors in favour of a surgical vs. conservative approach for our CNSL cohort are age < 80 years, ECOG PS < 2 and <2risk factors among age, ECOG PS, GCS score, location, multifocal or meningeal involvement. The results suggest a major contribution of the ECOG PS in the decision process, but also highlight the importance of a multifactorial assessment. The complication rate for our study cohort is smaller than what has been described in the literature (14.5% vs. 17.2%) [29], but higher when compared with risks of brain biopsies for other pathologies in general [30].

Aside from the procedural risks associated with the tissue diagnosis, sampling from a single tumour location does not account for the spatial inter- and intratumoral heterogeneity and would not reflect the dynamic genomic alterations in lymphoma such as the emergence of treatment-resistant clones post-therapy. Such limitations are prompting the need for the development of non-invasive technologies capable of capturing this heterogeneity such as liquid biopsies, which are currently used as methods of sampling and analysis of analytes from various biological fluids, believed to be more readily accessible than the standard tissue biopsy. In lymphoma, the analysis of tumour-circulating DNA (ctDNA) is the main liquid biopsy approach, and its applications have been best examined in DLBCL [31]. Several studies have demonstrated that ctDNA is more readily detectable from CSF vs. plasma in CNS lymphoma, as well as in primary and secondary brain tumours [31,32]. The detection of ctDNA in CSF has also been proven to be more sensitive than flow cytometry in detecting recurrent cases of both PCNSL and relapsed CNSL [31]. Another target for ctDNA analysis is the PCNSL tumour-specific MYD88 L265P mutation due to its presence in more than 80% of PCNSL tumours and very rare detection on other non-lymphoma CNS malignancies [31,32,33,34,35]. However, the identification of specific mutations in either blood or CSF of vitreous samples has various sensitivities, with studies suggesting that CSF sampling is more reliable for ctDNA measurements [31,32]. ctDNA levels in CSF have been used to assess the risk for CNS relapse, and one study finds that ctDNA mutations of five genes (BTG2, PIM1, DUSP2, ETV6 and CXCR4) are associated with high-risk CNS IPI scores [31,34]. The role of ctDNA in PCNSL or relapsed CNS lymphoma is less well-studied as compared with systemic DLBCL, and areas such as its concordance with tissue-based genotyping or its prognostic value require further exploration.

In terms of survival and outcome, one of the most commonly used risk models is the International Prognostic Index (IPI), which consists of five factors: age > 60 years, elevated lactate dehydrogenase (LDH), ECOG PS >1, advanced stage disease and involvement of more than one extranodal site [5,36]. The later-adopted CNS International Prognostic Index (CNS-IPI) consists of all of the previous factors with the addition of the kidney, adrenal, testis, pericardium, orbit and bone marrow involvement [5,13]. Apart from extranodal involvement, all of these risk factors are investigated in our CNSL cohort in relation to OS, but with a higher age threshold (80 years). In the multivariate regression, age is only found as a statistically significant risk factor for OS in the PCNSL group, whereas ECOG PS is a statistically significant factor for both PCNSL and the entire CNSL cohort. The disease stage and involvement of previously described high-risk sites are not found as significant risk factors for OS in our study. LDH is analysed alongside other two biochemical factors, decreased albumin, and elevated beta 2 microglobulin (B2M). Albumin is the only confirmed biochemical risk factor in the multivariate regression for PCNSL and the entire CNSL study group, and previously has been identified as risk factor for CNSL relapse [37].

The characterisation of DLBCL according to the cell of origin allows the distinction between the prognostically favourable germinal center B-cell (GC) and unfavourable activated B-cell (ABC) [1,13,29]. In our study, neither the cell of origin nor other molecular characteristics are found as risk factors for OS. DLBCL is the prevalent diagnosis, and ABC-like DLBCL subtype is more common among the PCNSL patients, in accordance with what has been previously described [10], as well as EBV positivity. Further characterisation of high-grade B-cell lymphoma achieved by next-generation sequencing, and mutations of MYC and BCL2 and/or BCL6 genes (“double-hit”-DHIT or “triple-hit”-THIT lymphoma) have been associated in various studies with a worse prognosis [1,2,13]. Our analysis does not find DHIT or THIT as risk factor; however, the sample size is small and therefore limits the data interpretation. Regarding the treatment for systemic disease, the lack of CNS prophylaxis is the only statistically significant risk factor for OS in the relapsed CNSL group. The role of CNS prophylaxis remains uncertain to this date, with no consensus regarding the optimal treatment or the selection of high-risk patients [1], and studies investigating the role of CNS prophylaxis have demonstrated heterogenous results [1,2,5,13]. Among the agents employed for CNSL relapse or PCNSL treatment, <3 cycles of systemic chemotherapy or not receiving ASCT are the risk factors for OS that retain statistical significance in the multivariate regression for both PCNSL and the entire CNSL group.

While prognostic scores designed to assess relapsed CNSL are generally addressing the risk of CNS recurrence, those employed for PCNSL evaluate the progression-free interval and the OS. To this date, four known disease prognostic models are used to estimate the PCNSL survival: the International Extranodal Lymphoma Study Group (IELSG), NB prognostic score, MSKCC prognostic model and the more recent Taipei score that has been validated in our study [17,36]. The IESLG includes five variables, namely age, ECOG PS, LDH, the level of cerebrospinal fluid (CSF) protein and deep brain involvement. The absence of CSF analysis and the incomplete availability of LDH results in our data prompts the exclusion of this score from our study analysis. The NB prognostic score employs three variables including age, ECOG PS and multifocal lesions or meningeal disease; the MSKCC includes age and Karnofsky PS; and the Taipei score utilises age, ECOG PS and deep brain involvement. The MSKCC prognostic model shows the best results when applied to the relapsed CNSL group but without reaching statistical significance in relation to OS, whereas the Taipei score has the best prognostic value for PCNSL and the entire CNSL population. Thus, our results support the use of the Taipei survival prognostic tool to support clinical evaluation and management of patients with CNS lymphoma.

## 5. Limitations

The retrospective nature of the study and the associated biases for data collection are the main limitations of this study. The variability of the sample sizes for the risk factors examined as well as the reduced number of cases underpin the need for cautious data interpretation.

## 6. Conclusions

CNS lymphoma remains an orphan disease with a poor survival outcome and an unmet treatment need that has been inconsistently addressed from a neurosurgical perspective. Despite data and methodological limitations, our study assesses the role of a multidisciplinary neuro-oncology team in the management of CNSL. The MDT is effective in selecting those requiring neurosurgical intervention whilst avoiding the risks where CNS biopsy has no added value or risks are deemed unacceptable. Despite advances in therapy, the OS for this group of patients remains limited, further emphasising the need to minimise risks to allow the extension of a good quality of life for as long as possible.

## Figures and Tables

**Table 1:** Clinical characteristics of the CNSL surgical and non-surgical cases.

		CNSL Surgical Cases		CNSL Non-Surgical Cases
	Total	Biopsy	Resection	Total
Age < 80	71/76 (93.4%)	64/66 (96.9%)	10/10 (100%)	41/53 (77.3%)
ECOG PS < 2	65/76 (85.5%)	57/66 (86.3%)	10/10 (100%)	18/53 (33.9%)
GCS 15/15	61/76 (80.2%)	55/66 (83.3%)	8/10 (80%)	36/53 (67.9%)
Symptoms at presentation				
Neurological deficit	49/76 (64.4%)	43/66 (65.1%)	6/10 (60%)	37/53 (69.8%)
Seizures	9/76 (11.8%)	7/66 (10.6%)	2/10 (20%)	1/53 (1.8%)
Cognitive impairment	26/76 (34.2%)	21/66 (31.8%)	5/10 (50%)	14/53 (26.4%)
Comorbidities *				
HIV	4/76 (5.2%)	3/66 (4.5%)	1/10 (10%)	3/53 (5.6%)
Malignant diseases	10/76 (13.1%)	8/66 (12.1%)	2/10 (20%)	4/53 (7.5%)

* Cardiovascular and respiratory comorbidities are analysed in Table 5.

**Table 2:** Summary of the neuro-oncology MDT diagnoses for the cases that underwent neurosurgery (biopsy or resection)—surgical cases, and those who did not undergo neurosurgery—non-surgical cases. The likelihood ratio has been displayed for the surgical cases for those where lymphoma was considered as single or top differential diagnosis, and for the cases where lymphoma was considered either single, first or among the differential diagnoses. Positive predictive values (PPV) displayed for each diagnostic category.

	Total	Lymphoma as Single/Top Diagnosis		Lymphoma among Differential Diagnoses	
		No	Yes	No	Yes
Surgical cases (total)	76	30	46	15	61
Confirmed	74	30/30	44/46	15/15	59/61
Not-confirmed	2	-	2/46	-	2/61
Likelihood ratio (*p* value)		2.044 (*p* = 0.153)		0.893 (*p* = 0.345)	
Non-surgical cases (total)	53	6	47	4	49
Confirmed *	8	-	8/47	-	8/49
Not-confirmed *	2	-	2/47	-	2/49
True not-confirmed	43	6/6	37/47	4/4	39/49
PPV		95.6%		96.7%	

Note: PPV—positive predictive value; True not-confirmed—no tissue diagnosis performed (brain or extracranial). * non-brain tissue histopathological diagnosis.

**Table 3:** Summary of the neuro-oncology MDT diagnoses for the cases of relapsed CNSL and PCNSL. Those who underwent neurosurgery and had a histopathological confirmation of lymphoma are referred to as confirmed cases, and those who did not as not-confirmed cases. Positive predictive values (PPV) displayed for the cases where lymphoma was considered as single or first differential diagnosis, and for the cases where lymphoma was considered either single, first or among the differential diagnoses. The likelihood ratio has been displayed for PCNSL for each diagnostic category.

	Total	Total among Surgical Cases	Confirmed Cases	Not-Confirmed Cases	PPV	Likelihood Ratio	*p* Value
Relapsed CNSL	36						
Biopsy/Resection							
Yes	15		15/15	-			
No	21		7 */21	14/21			
Lymphoma as single/first diagnosis					100%	-	-
Yes	33	15	12/15	-			
No	3	-	3/15	-			
Lymphoma among differential diagnoses					100%	-	-
Yes	34	15	13/15	-			
No	2	-	2/15	-			
PCNSL	93						
Biopsy/Resection							
Yes	61		59/61	2/61			
No	32		1 */32	2 †/32			
Lymphoma as single/first diagnosis					97%	2.392	0.122
Yes	61	34	32/61	2/61			
No	32	27	27/61	-			
Lymphoma among differential diagnoses					95.8%	0.977	0.323
Yes	75	48	46/61	2/61			
No	18	13	13/61	-			

* Confirmed via non-brain biopsy; † Not-confirmed via CSF analysis.

**Table 4:** Summary of the characteristics of the surgical cases where histopathology or CSF analysis did not confirm lymphoma.

Non-Confirmed Cases	Lymphoma as Single/First Diagnosis	Category	Comorbidities	Location
Pineocytoma grade I	yes	PCSNL	HIV	3rd ventricle
Diffuse astrocytoma gr II	yes	PCNSL	-	Corpus callosum, brainstem
Toxoplasmosis *	yes	PCNSL	HIV concomitant diagnosis	Periventricular, ependymal, leptomeningeal
EBV infection *	yes	PCNSL	Primary myelofibrosis	Basal ganglia

* CSF analysis.

## Data Availability

All data relevant to the study are included in the article or upoaded as Supplementary Information.

## Supplementary Materials

The following supporting information can be downloaded at: https://www.mdpi.com/article/10.3390/jpm13050783/s1. Table S1: Summary of the risk factors for the OS in the groups of relapsed and primary CNS lymphoma, and their hazard ratios (HR) displayed for the univariate and multivariate analysis. Note: CI—confidence interval; LDH—lactate dehydrogenase; B2M—beta-2 macroglobulin; HD-MTX—high dose methotrexate. Table S2:. Summary of the risk factors for OS for the entire cohort of CNS lymphoma, and their hazard ratios (HR) displayed for the univariate and multivariate analysis. Note: HR—hazard ratio; CI—confidence interval; LDH—lactate dehydrogenase; B2M—beta-2 macroglobulin; HD-MTX—high dose methotrexate. Legend * Lymphoma affecting sites previously described at high risk of CNS recurrence † Systemic high dose methotrexate. Table S3: Summary of Median and Mean OS of the Relapsed CNSL, PCSNL and the entire CNSL cohorts expressed in months for each prognostic score used in the study. Note: * sample size of 2.
