# Supplementary material for: Neurosurgical Management of Central Nervous System Lymphoma: Lessons Learnt from a Neuro-Oncology Multidisciplinary Team Approach
Source: J Pers Med. 2023 Apr 30;13(5):783. doi: 10.3390/jpm13050783 (PMC10221289; doi:10.3390/jpm13050783)
Supplement: Supplementary file 1 [file jpm-13-00783-s001.zip › Table S1.pptx]

## Slide 1
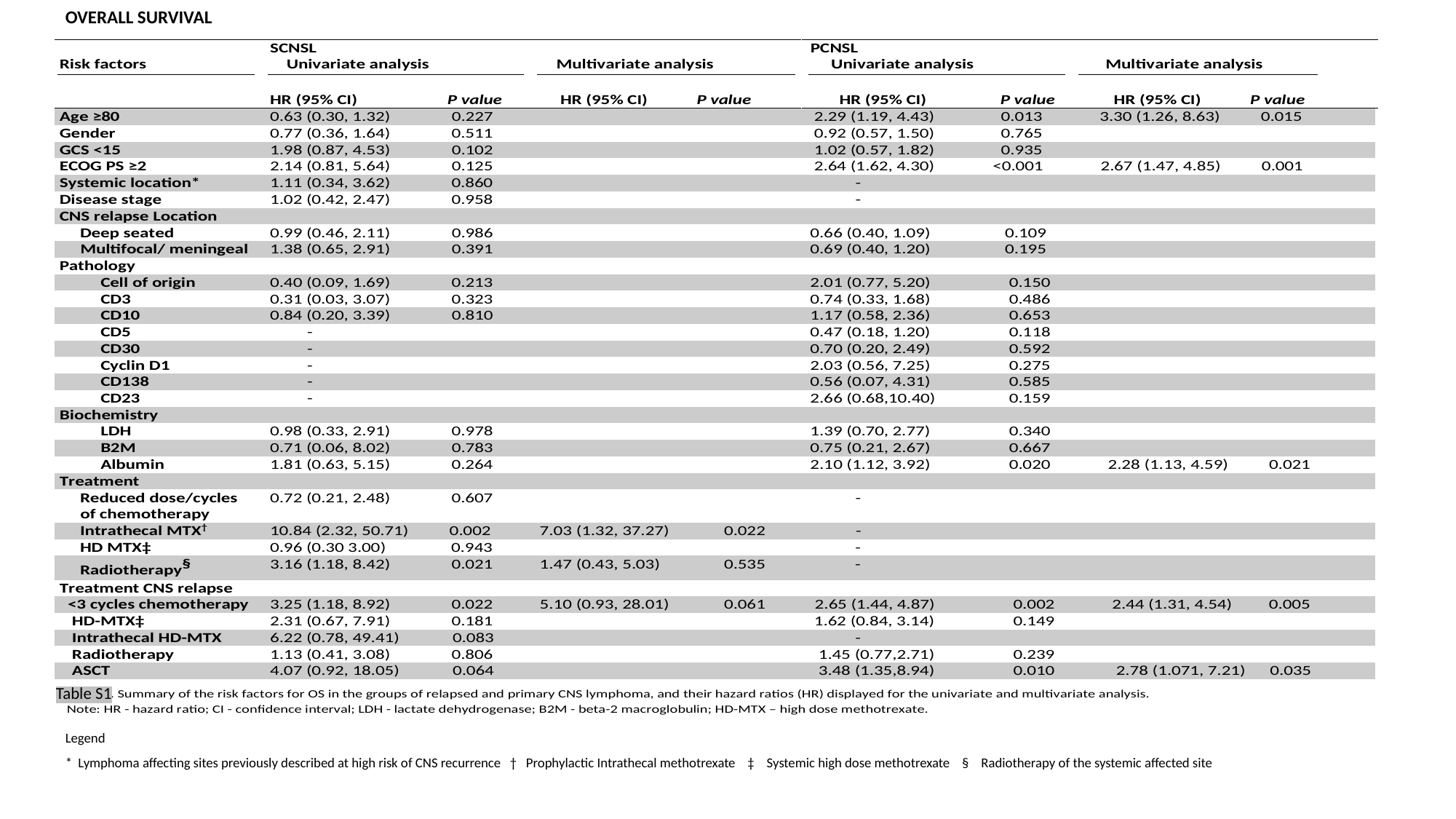

OVERALL SURVIVAL
Table S1
Legend
* Lymphoma affecting sites previously described at high risk of CNS recurrence † Prophylactic Intrathecal methotrexate ‡ Systemic high dose methotrexate § Radiotherapy of the systemic affected site
