# Supplementary material for: Neurosurgical Management of Central Nervous System Lymphoma: Lessons Learnt from a Neuro-Oncology Multidisciplinary Team Approach
Source: J Pers Med. 2023 Apr 30;13(5):783. doi: 10.3390/jpm13050783 (PMC10221289; doi:10.3390/jpm13050783)
Supplement: Supplementary file 1 [file jpm-13-00783-s001.zip › Table S2 .pptx]

## Slide 1
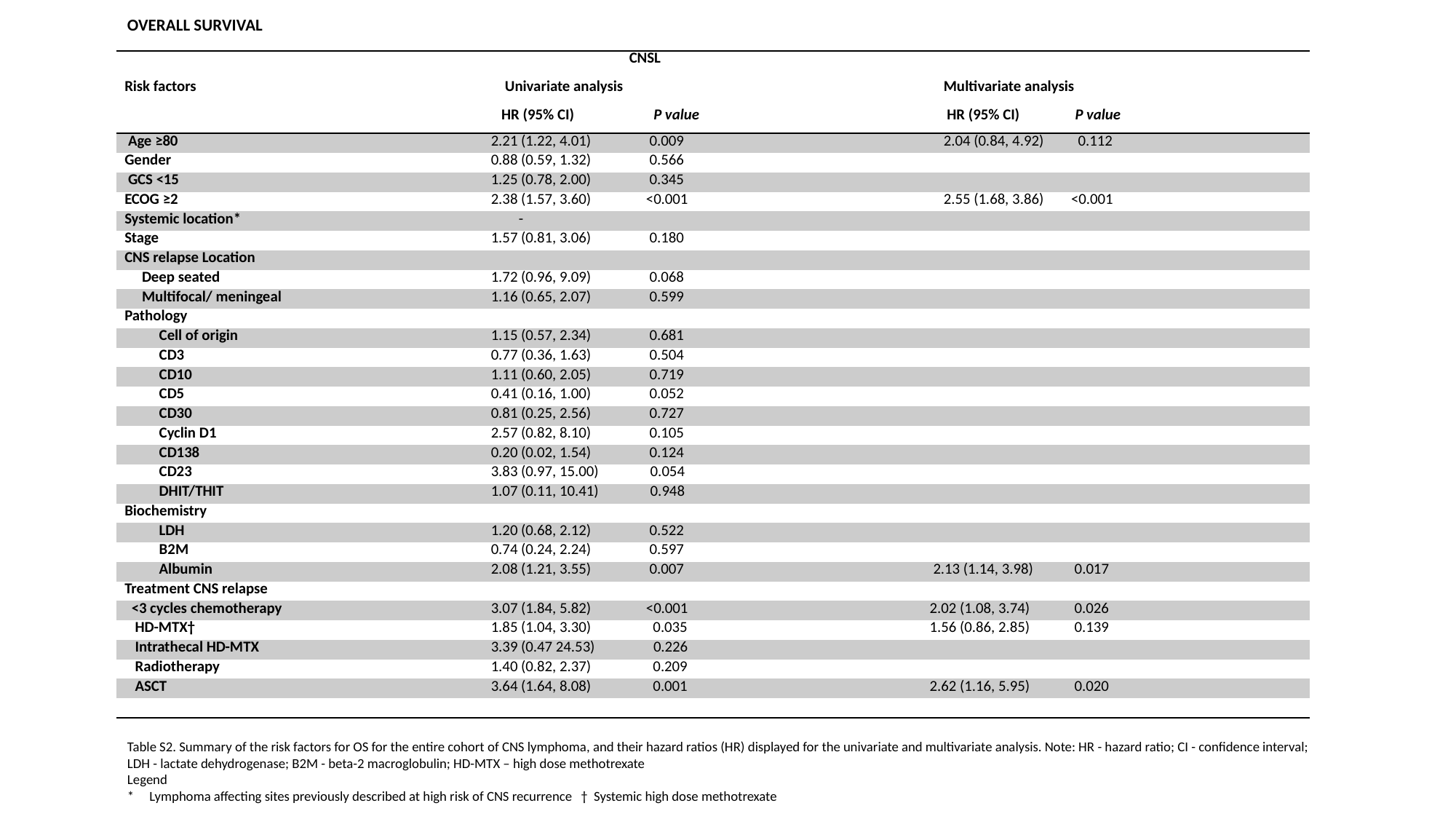

OVERALL SURVIVAL
| Risk factors | CNSL Univariate analysis HR (95% CI) P value | Multivariate analysis HR (95% CI) P value |
| --- | --- | --- |
| Age ≥80 | 2.21 (1.22, 4.01) 0.009 | 2.04 (0.84, 4.92) 0.112 |
| Gender | 0.88 (0.59, 1.32) 0.566 | |
| GCS <15 | 1.25 (0.78, 2.00) 0.345 | |
| ECOG ≥2 | 2.38 (1.57, 3.60) <0.001 | 2.55 (1.68, 3.86) <0.001 |
| Systemic location\* | - | |
| Stage | 1.57 (0.81, 3.06) 0.180 | |
| CNS relapse Location | | |
| Deep seated | 1.72 (0.96, 9.09) 0.068 | |
| Multifocal/ meningeal | 1.16 (0.65, 2.07) 0.599 | |
| Pathology | | |
| Cell of origin | 1.15 (0.57, 2.34) 0.681 | |
| CD3 | 0.77 (0.36, 1.63) 0.504 | |
| CD10 | 1.11 (0.60, 2.05) 0.719 | |
| CD5 | 0.41 (0.16, 1.00) 0.052 | |
| CD30 | 0.81 (0.25, 2.56) 0.727 | |
| Cyclin D1 | 2.57 (0.82, 8.10) 0.105 | |
| CD138 | 0.20 (0.02, 1.54) 0.124 | |
| CD23 | 3.83 (0.97, 15.00) 0.054 | |
| DHIT/THIT | 1.07 (0.11, 10.41) 0.948 | |
| Biochemistry | | |
| LDH | 1.20 (0.68, 2.12) 0.522 | |
| B2M | 0.74 (0.24, 2.24) 0.597 | |
| Albumin | 2.08 (1.21, 3.55) 0.007 | 2.13 (1.14, 3.98) 0.017 |
| Treatment CNS relapse | | |
| <3 cycles chemotherapy | 3.07 (1.84, 5.82) <0.001 | 2.02 (1.08, 3.74) 0.026 |
| HD-MTX† | 1.85 (1.04, 3.30) 0.035 | 1.56 (0.86, 2.85) 0.139 |
| Intrathecal HD-MTX | 3.39 (0.47 24.53) 0.226 | |
| Radiotherapy | 1.40 (0.82, 2.37) 0.209 | |
| ASCT | 3.64 (1.64, 8.08) 0.001 | 2.62 (1.16, 5.95) 0.020 |
| | | |
Table S2. Summary of the risk factors for OS for the entire cohort of CNS lymphoma, and their hazard ratios (HR) displayed for the univariate and multivariate analysis. Note: HR - hazard ratio; CI - confidence interval; LDH - lactate dehydrogenase; B2M - beta-2 macroglobulin; HD-MTX – high dose methotrexate
Legend
* Lymphoma affecting sites previously described at high risk of CNS recurrence † Systemic high dose methotrexate
