# Supplementary material for: Neurosurgical Management of Central Nervous System Lymphoma: Lessons Learnt from a Neuro-Oncology Multidisciplinary Team Approach
Source: J Pers Med. 2023 Apr 30;13(5):783. doi: 10.3390/jpm13050783 (PMC10221289; doi:10.3390/jpm13050783)
Supplement: Supplementary file 1 [file jpm-13-00783-s001.zip › Table S3 .pptx]

## Slide 1
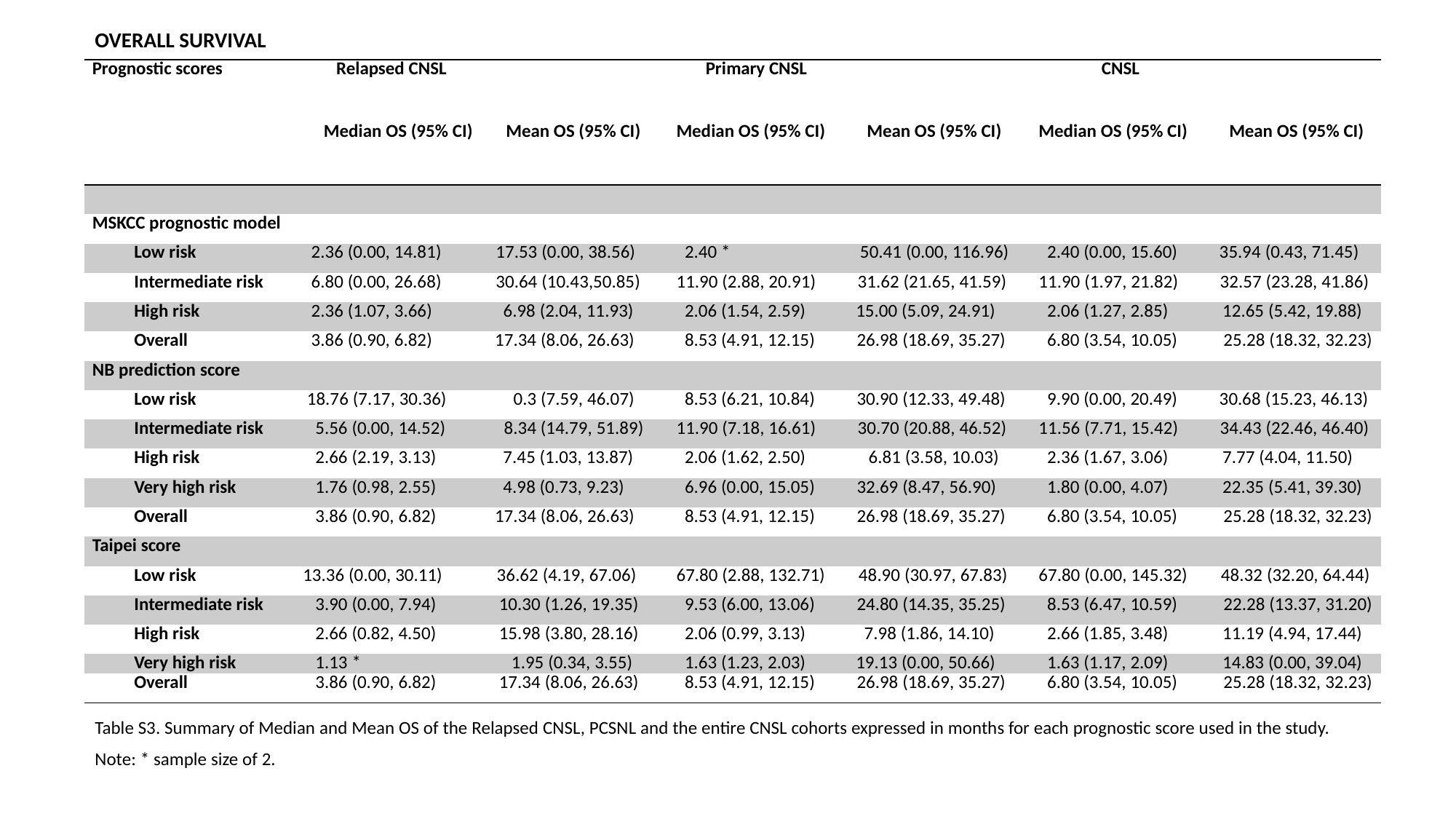

OVERALL SURVIVAL
| Prognostic scores | Relapsed CNSL   Median OS (95% CI) Mean OS (95% CI) | Primary CNSL   Median OS (95% CI) Mean OS (95% CI) | CNSL   Median OS (95% CI) Mean OS (95% CI) |
| --- | --- | --- | --- |
| | | | |
| MSKCC prognostic model | | | |
| Low risk | 2.36 (0.00, 14.81) 17.53 (0.00, 38.56) | 2.40 \* 50.41 (0.00, 116.96) | 2.40 (0.00, 15.60) 35.94 (0.43, 71.45) |
| Intermediate risk | 6.80 (0.00, 26.68) 30.64 (10.43,50.85) | 11.90 (2.88, 20.91) 31.62 (21.65, 41.59) | 11.90 (1.97, 21.82) 32.57 (23.28, 41.86) |
| High risk | 2.36 (1.07, 3.66) 6.98 (2.04, 11.93) | 2.06 (1.54, 2.59) 15.00 (5.09, 24.91) | 2.06 (1.27, 2.85) 12.65 (5.42, 19.88) |
| Overall | 3.86 (0.90, 6.82) 17.34 (8.06, 26.63) | 8.53 (4.91, 12.15) 26.98 (18.69, 35.27) | 6.80 (3.54, 10.05) 25.28 (18.32, 32.23) |
| NB prediction score | | | |
| Low risk | 18.76 (7.17, 30.36) 0.3 (7.59, 46.07) | 8.53 (6.21, 10.84) 30.90 (12.33, 49.48) | 9.90 (0.00, 20.49) 30.68 (15.23, 46.13) |
| Intermediate risk | 5.56 (0.00, 14.52) 8.34 (14.79, 51.89) | 11.90 (7.18, 16.61) 30.70 (20.88, 46.52) | 11.56 (7.71, 15.42) 34.43 (22.46, 46.40) |
| High risk | 2.66 (2.19, 3.13) 7.45 (1.03, 13.87) | 2.06 (1.62, 2.50) 6.81 (3.58, 10.03) | 2.36 (1.67, 3.06) 7.77 (4.04, 11.50) |
| Very high risk | 1.76 (0.98, 2.55) 4.98 (0.73, 9.23) | 6.96 (0.00, 15.05) 32.69 (8.47, 56.90) | 1.80 (0.00, 4.07) 22.35 (5.41, 39.30) |
| Overall | 3.86 (0.90, 6.82) 17.34 (8.06, 26.63) | 8.53 (4.91, 12.15) 26.98 (18.69, 35.27) | 6.80 (3.54, 10.05) 25.28 (18.32, 32.23) |
| Taipei score | | | |
| Low risk | 13.36 (0.00, 30.11) 36.62 (4.19, 67.06) | 67.80 (2.88, 132.71) 48.90 (30.97, 67.83) | 67.80 (0.00, 145.32) 48.32 (32.20, 64.44) |
| Intermediate risk | 3.90 (0.00, 7.94) 10.30 (1.26, 19.35) | 9.53 (6.00, 13.06) 24.80 (14.35, 35.25) | 8.53 (6.47, 10.59) 22.28 (13.37, 31.20) |
| High risk | 2.66 (0.82, 4.50) 15.98 (3.80, 28.16) | 2.06 (0.99, 3.13) 7.98 (1.86, 14.10) | 2.66 (1.85, 3.48) 11.19 (4.94, 17.44) |
| Very high risk | 1.13 \* 1.95 (0.34, 3.55) | 1.63 (1.23, 2.03) 19.13 (0.00, 50.66) | 1.63 (1.17, 2.09) 14.83 (0.00, 39.04) |
| Overall | 3.86 (0.90, 6.82) 17.34 (8.06, 26.63) | 8.53 (4.91, 12.15) 26.98 (18.69, 35.27) | 6.80 (3.54, 10.05) 25.28 (18.32, 32.23) |
Table S3. Summary of Median and Mean OS of the Relapsed CNSL, PCSNL and the entire CNSL cohorts expressed in months for each prognostic score used in the study.
Note: * sample size of 2.
